# CT features associated with EGFR mutations and ALK positivity in patients with multiple primary lung adenocarcinomas

**DOI:** 10.1186/s40644-020-00330-1

**Published:** 2020-07-20

**Authors:** Xiaoyu Han, Jun Fan, Jin Gu, Yumin Li, Ming Yang, Tong Liu, Nan Li, Wenjuan Zeng, Heshui Shi

**Affiliations:** 1 Department of Radiology, Union Hospital, Tongji Medical College, Huazhong University of Science and Technology, 1277 Jiefang Rd, Wuhan, Hubei Province 430022 People’s Republic of China; 2 Department of Pathology, Union Hospital, Tongji Medical College, Huazhong University of Science and Technology, 1277 Jiefang Rd, Wuhan, Hubei Province 430022 People’s Republic of China; 3 Department of Clinical Laboratory, Union Hospital, Tongji Medical College, Huazhong University of Science and Technology, Wuhan, 430022 China

**Keywords:** Adenocarcinoma, Epidermal growth factor receptor, Anaplastic lymphoma kinase, X-ray computed tomography

## Abstract

**Background:**

In multiple primary lung adenocarcinomas (MPLAs), the relationship between imaging and gene mutations remains unclear. This retrospective study aimed to identify the correlation of epidermal growth factor receptor (EGFR) mutations and anaplastic lymphoma kinase (ALK) status with CT characteristics in MPLA patients.

**Methods:**

Sixty-seven patients (135 lesions) with MPLAs confirmed by pathology were selected from our institution. All subjects were tested for EGFR mutations and ALK status and underwent chest CT prior to any treatment. The criteria for MPLA definitions closely adhered to the comprehensive histologic assessment (CHA).

**Results:**

Among MPLA patients, EGFR mutations were more common in females (*p* = 0.002), in those who had never smoked (*p* = 0.010), and in those with less lymph node metastasis (*p* < 0.001), and the tumours typically presented with ground-glass opacity (GGO) (*p* = 0.003), especially mixed GGO (*p* < 0.001), and with air bronchograms (*p* = 0.012). Logistics regression analysis showed that GGO (OR = 6.550, *p* = 0.010) was correlated with EGFR mutation, while air bronchograms were not correlated with EGFR mutation (OR = 3.527, *p* = 0.060). A receiver operating characteristic (ROC) curve yielded area under the curve (AUC) values of 0.647 and 0.712 for clinical-only or combined CT features, respectively, for prediction of EGFR mutations, and a significant difference was found between them (*p* = 0.0344). ALK-positive status was found most frequently in MPLA patients who were younger (*p* = 0.002) and had never smoked (*p* = 0.010). ALK positivity was associated with solid nodules or masses in MPLAs (*p* < 0.004) on CT scans. Logistics regression analysis showed that solid nodules (OR = 6.550, *p* = 0.010) were an independent factor predicting ALK positivity in MPLAs. For prediction of ALK positivity, the ROC curve yielded AUC values of 0.767 and 0.804 for clinical-only or combined CT features, respectively, but no significant difference was found between them (*p* = 0.2267).

**Conclusion:**

Among MPLA patients, nonsmoking women with less lymph node metastasis and patients with lesions presenting GGO or mixed GGO and air bronchograms on CT were more likely to exhibit EGFR mutations. In nonsmoking patients, young patients with solid lesions on CT are recommended to undergo an ALK status test.

## Introduction

Lung cancer has been a leading cause of cancer-related death worldwide for decades. In 1975, Manini and Melamed first introduced the concept and diagnostic criteria for multiple primary lung cancers (MPLCs) [1, 2]. The proportion of adenocarcinoma in MPLCs is much higher than that of squamous cell carcinoma. With the widespread use of computed tomography (CT) and lung cancer screening, the incidence of MPLAs in patients has been reported as 0.2 to 8% (3.5 to 14% in autopsy studies) [3–5]. Although there is no standard for MPLA treatment, the consensus is that different lesions should be managed and staging separately [6, 7]. At present, surgical resection is still the main option for treatment of MPLAs [8, 9]. However, considering a patient’s tolerance, it is difficult to resect all MPLA lesions. Randomized clinical trials have demonstrated that in advanced non-small cell lung cancer (NSCLC) progression-free survival (PFS) is longer following treatment with tyrosine kinase inhibitors (TKIs) than following chemotherapy [10, 11]. Therefore, from the perspective of treatment, genetic testing is still needed for MPLA lesions.

Epidermal growth factor receptor (EGFR) mutations and anaplastic lymphoma kinase (ALK) rearrangement are the two most common druggable targets in lung adenocarcinoma. Recent studies have found that driver mutations (including EGFR and ALK mutations) are highly inconsistent among MPLCs [12, 13], emphasizing the need to separately analyse gene mutation status in multifocal tumours [6]. However, obtaining sufficient tissue from multifocal lung adenocarcinomas for gene mutation analysis before treatment may not be feasible due to inoperability, sampling artefacts or limited biopsy specimen amounts. Therefore, more convenient, noninvasive approaches are needed to augment gene status assessments in patients with nonresectable, multifocal lung adenocarcinomas.

CT is the optimal method to detect and characterize pulmonary tumours. The association between CT features and EGFR mutation or ALK positivity in single primary lung adenocarcinoma (SPLA) has been well established [14–21] and indicates a certain correlation between imaging and gene expression in SPLA. However, to the best of our knowledge, no previous study of MPLAs has evaluated the association of EGFR and ALK mutations with CT features. Therefore, our study aims to explore whether CT characteristics can predict EGFR mutations and ALK positivity in patients with MPLAs.

## Materials and methods

### Patients and inclusion criteria

A total of 1193 patients evaluated by the multidisciplinary thoracic oncology group between January 2014 and February 2019 at the Union Hospital of Tongji Medical College were retrospectively screened. Among them, 107 patients (235 lesions) with at least 2 or more synchronous multiple pulmonary adenocarcinomas according to comprehensive histologic assessment (CHA) [2, 22] during an initial thin-section CT scan were retrospectively evaluated in this study. The inclusion criteria were as follows: (a) pathologically confirmed by surgical resection as lung adenocarcinoma; (b) available pathology reports (including predominant pathological subtype, lymph node metastasis and pleural invasion) with a diagnosis of lung adenocarcinoma; (c) available results for both EGFR mutations and ALK status; and (d) available clinical data, including age, sex, smoking history, and tumour node metastasis (TNM) stage. The exclusion criteria were as follows: (a) thin-section CT was not available (*n* = 21); (b) the time interval between CT acquisition and surgery was more than 3 months (*n* = 10); (c) preoperative treatment prior to surgery, such as radiation therapy or chemotherapy (*n* = 6); and (d) CT image artefacts that precluded further evaluation (*n* = 3). A total of 67 patients (135 lesions, with 103 synchronous and 22 metachronous lesions) were ultimately included. The medical records of each patient were reviewed retrospectively. Patient clinical characteristics, including age, sex, smoking history, histopathology, nodal involvement, and tumour stage, were recorded. In accordance with Lv et al. [23], nonsmoking was defined as lifetime exposure to fewer than 100 cigarettes, and the remaining patients were categorized as ever-smokers. TNM staging was based on the IASLC 8th TNM Lung Cancer Staging System [7]. When staging tumours according to the current staging system was difficult, a multidisciplinary team (MDT) was organized to discuss and reach a consensus [24]. This retrospective study was approved by the Institutional Review Board of Union Hospital of Tongji Medical College. All subjects enrolled in this study signed a written consent form after being informed of the research details.

Notably, the present study was conducted on a per lesion basis or per patient basis in different situations as follows: clinical characteristics (age, sex, smoking history and lymph node metastasis) were described on a per patient basis. EGFR mutations in an MPLA patient were considered if at least one lesion harboured an EGFR mutation. Similarly, ALK-positive MPLA patients were considered if at least one lesion harboured an ALK-positive lesion. On the other hand, the tumour TNM stage, CT findings, EGFR status, and ALK status were described for each lesion.

### MPLA Histopathological analysis and evaluation criteria

The predominant subtype of lung adenocarcinoma was assessed according to the IASLC/ATS/ERS classification of lung adenocarcinoma. Pathological subtypes of nodules were recorded as the predominant pattern, including atypical adenoma-like hyperplasia (AAH); adenocarcinoma in situ (AIS); microinvasive adenocarcinoma (MIA); invasive adenocarcinoma with predominant lepidic, acinar, papillary, solid, or micropapillary components; and invasive adenocarcinoma variants (including mucinous, foetal, and enteric). Adenocarcinomas with lepidic patterns were defined as minimally invasive adenocarcinomas, adenocarcinomas in situ or invasive adenocarcinomas with lepidic components. Designation of MPLAs in the present study was based on the CHA [2, 22]. MPLAs were histologically indicated as follows: (1) at least one of the multiple lesions was AIS or MIA; (2) the predominant histopathologic pattern was different between multiple lesions; (3) the predominant histopathologic pattern was similar, but there were differences in EGFR or ALK status between multiple lesions; (4) synchronous MPLAs were defined when the tumour-free interval between cancers was < 2 years, and metachronous lesions were defined when the tumour-free interval between cancers was ≥2 years.

### EGFR mutation analysis

EGFR mutations were analysed with the amplification-refractory mutation system (ARMS). Primary tumours, lymph nodes, distant metastases, and pleural effusion specimens were excised, aspirated, or biopsied; fixed in 10% neutral buffered formalin; and then embedded in paraffin. DNA was extracted from the formalin-fixed, paraffin-embedded tissue sections, and a Qiagen FFPE Tissue Kit (Netherlands Roots NV) was used according to the manufacturer’s instructions. PCR was carried out with the Mx3000PtM system (Stratagene, La Jolla, USA) using an EGFR 29 Mutations Detection Kit (Amoy Diagnostics, Xiamen, People’s Republic of China), and the results were interpreted according to the manufacturer’s instructions. Molecular analysis of EGFR mutations was defined as the mutation status of EGFR exons 18, 19,21, 20. Otherwise, other types of EGFR mutations were defined as wild-type EGFR [4, 17].

### VENTANA ALK immunohistochemistry (IHC) assay

The VENTANA IHC assay is a fully automated detection method based on the monoclonal antibody D5F3. This assay has been approved by the US FDA and China FDA for ascertaining the eligibility of patients with NSCLC for treatment with ALK TKIs. Formalin-fixed, paraffin-embedded tissue sections with a thickness of 4 μm were cut according to the manufacturer’s instructions and scored according to the supplied algorithm. The results were dichotomous, where any percentage of positive tumour cells with strong granular cytoplasmic staining indicated ALK positivity, while all other observations were regarded as ALK negativity.

### CT image acquisition

All 67 patients (135 lesions) underwent nonenhanced CT scanning, and 52 patients (105 lesions) also underwent enhanced CT scanning. CT imaging was performed at our institution using a multislice spiral CT system (SOMATOM Definition AS +, Siemens Healthineers, Germany). The scan ranged from the chest inlet to the inferior level of the costophrenic angle. The CT parameters were as follows: detector collimation width, 64 × 0.6 mm and 128 × 0.6 mm; tube voltage, 120 kV. The tube current was regulated by an automatic exposure control system (CARE Dose 4D). Images were reconstructed with a slice thickness of 1.5 mm and an interval of 1.5 mm. Then, the reconstructed image was transmitted to the workstation and picture archiving and communication systems (PACS) for multiplanar reconstruction (MPR) post-processing. The mediastinal window (centre, 50; width, 350) and lung window (centre, − 600; width, 1200) were obtained from the PACS. A nonionic iodine contrast agent (60–80 mL iohexol 350 mg/mL; Beilu Pharmaceutical Co., Ltd., Beijing, China) was applied to 52 patients via intravenous injection in the elbow, and the dose was 3 mL/s.

### CT image interpretation

Images were analysed by two radiologists (J.G., thoracic radiologist with 10 years of experience and X. H, a radiology fellow with 4 years of experience in interpreting CT images). Both radiologists used the Digital Imaging and Communications in Medicine (DICOM) protocol to analyse images from the CT studies without access to clinical and histologic findings but were aware of the presence and sites of the tumours. They assessed the CT features using both axial CT images and MPR images. After they performed separate evaluations, the differences were resolved by consensus. Interpretations of each CT characteristic are presented in Table 1.

**Table 1:** CT features for lung adenocarcinoma

Variable	Definition
Type	Central, tumour located in the segmental or more proximal bronchi; peripheral, tumour located in the subsegmental bronchi or more distal airway
Location	The distribution of each lesion in the lung was recorded, including left upper lobe, left lower lobe, right upper lobe, right middle lobe and right lower lobe
Relationship of location between multiple lesions in same patient	Same lung segment, same lung lobe, ipsilateral lung and heterolateral lung
Tumor size	Longest diameter of the tumor in MPR images
Ground glass opacity	Ground glass dense nodules with internal vessels and bronchi visible
Mix ground glass opacity	Composition of both ground glass opacity and solid
Pure ground glass opacity	Composition of ground glass opacity only
Texture	Predominantly solid, Tumour solid component / ground glass component > 0.5; Predominantly ground glass opacity, tumour solid diameter / ground glass diameter ≤ 0.5
Shape	indicated as lobulated, others (round, or oval)
Lobulated	The surface of the tumor showed as multiple arc-shaped projections
Margin	Evaluated in the lung window, and indicated as smooth, or spiculated
Spiculate	Evaluated in the lung window, and indicated as different degrees of spinous or burr-like protrusions at the tumour margin
Margin definition	evaluated in the lung window, and indicated as well-defined, or poor-defined
Air bronchogram	Tubelike or branched air structure within the tumour
Bubble-like lucency	The 1 ~ 3 mm of air density area within the mass
Margins	Evaluated in the lung window, and indicated as smooth, or spiculated
Heterogeneity	Evaluated in the soft tissue window, and heterogeneity indicated as the difference of CT values in tumor was greater than 20HU
Pleural attachment	Retraction of the pleura toward the tumour
Cavitation	Presence or absence of cavitation
Intramodular calcifications	Presence or absence of calcifications
Necrosis	Low-density area in the tumour, without enhancement in enhance CT
Peripheral emphysema	Presence or absence of peripheral emphysema
Peripheral fibrosis	Pulmonary fibrosis around the tumor
Vascular convergence	Convergence of vessels to the tumor, applied to the peripheral tumors
Enhancement	mild” = 0 ~ 20 HU; “moderate” = 20 ~ 40 HU, “marked” > 40 HU
Lymphadenopathy	presence or absence of lymphadenopathy thoracic lymph nodes (hilar or mediastinal) with short-axis diameter greater than 1 cm

### Statistical analysis

Analyses were performed using SPSS Statistics (SPSS, version 21, IBM, Chicago, IL, USA) and MedCalc 16.2.0 (MedCalc Software, Mariakerke, Belgium) software. Clinical characteristics (age, sex, smoking history and lymph node metastasis) are described on a per patient basis. Other clinical and pathological findings, CT features, EGFR status, and ALK status descriptions are described on a per lesion basis. The normality of the distribution was checked using a Kolmogorov-Smirnov test. Normally and nonnormally distributed data and categorical variables are expressed as the mean ± standard deviation, median (interquartile range) and frequency (percentage), respectively. An independent-sample Student’s *t* test was used to compare two groups of normally distributed variables, and a chi-square test was used to compare categorical variables. Multiple logistic regression analyses were performed to identify independent factors predictive of EGFR or ALK mutation status. The final model was selected by using the enter elimination method, with a cutoff *p* value of 0.05. A *p* value < 0.05 (two-tailed) was considered to be statistically significant. Receiver operating characteristic curves (ROCs) were constructed for the combined independent factors for predicting EGFR mutations or ALK positivity. Then, a comparison of ROC curves between clinical characteristics alone and clinical characteristics combined with CT signs was performed using the nonparametric approach of DeLong et al. The repeatability test of tumour maximum diameter was analysed using the intraclass correlation coefficient (ICC) with a 95% confidence interval (CI). For other CT signs, interobserver agreement was assessed with the *k* coefficient [25]. A *p* value < 0.05 (two-tailed) was considered to be statistically significant.

## Results

### Clinical characteristics

The incidence of MPLAs was 5.6% (67/1193) in our hospital from January 2014 to February 2019. A total of 67 eligible MPLA patients (58 ± 7 years, ranging from 35 to 73 years; female/male ratio: 2/1) were enrolled, including 135 lesions. In total, 26.9% of MPLA patients were smokers.

### Correlations of EGFR mutations and ALK status with clinical features

When based on patients (*n* = 67), subjects were divided into an EGFR mutation (*n* = 43) group and a wild-type EGFR group (*n* = 24). As shown in Table 2, EGFR mutations were found more frequently in females (*p* = 0.011), those who had never smoked (*p* = 0.041), and those with less lymph node metastasis (*p* < 0.001), but no significant association was found with age (56 ± 6 vs 60 ± 8 years, *p* = 0.068). When based on lesions (*n* = 135), no differences were found between the EGFR mutation (*n* = 43) group and the wild-type EGFR (*n* = 24) group in terms of TNM stage, pathological subtype or pleural invasion, as shown in Table 3.

**Table 2:** Clinical comparison of multiple primary lung adenocarcinomas in different EGFR and ALK status (in pre-patients)

Variable	EGFR+ (*n* = 43)	EGFR- (*n* = 24)	Total	*P*	ALK+ (*n* = 13)	ALK-(n = 54)	Total	*P*
Age	56 ± 6	60 ± 8	58 ± 7	0.068	49 ± 7	58 ± 7	58 ± 7	0.002*
Gender				0.011*				0.099
Male	10(23)	13(54)	23(34)		7(54)	16(30)	23(34)	
Female	33(77)	11(46)	44(66)		6(46)	35(4)	44(66)	
History of smoking	8(19)	10(42)	18(27)	0.041*	2(14)	16(30)	18(27)	0.010*
Lymph node metastasis	10(23)	16(67)	26(30)	<0.001*	5(38)	8(15)	26(39)	0.110

* *P* values were based on comparisons between the two groups

*EGFR* epidermal growth factor receptor, *ALK* anaplastic large-cell lymphoma kinase

EGFR +, EGFR mutation; EGFR-, EGFR wild type mutation; ALK +, ALK positive; ALK-, ALK negative

**Table 3:** Pathology comparison of of multiple primary lung adenocarcinomas in different EGFR and ALK status (in pre-lesions)

Variable	EGFR+ (*n* = 62)	EGFR- (*n* = 73)	Total	*P*	ALK+ (*n* = 23)	ALK-(*n* = 112)	Total	*P*
TNM stage^a^				0.312				0.160
I-II	22(35)	20(27)	42(31)		10(43)	32(29)	42(31)	
III-IV	40(65)	53(73)	93(29)		13(57)	80(71)	93(68)	
Histological subtype								
Lepidic predominant^b^	15(24)	10(14)	25(19)	0.118	3(13)	22(20)	25(19)	0.567
other subtype1^c^	47(76)	63(86)	110(81)		20(87)	90(80)	110(81)	
Solid or Mucinous	5(8)	11(15)	16(12)	0.210	8(35)	8(7)	16(12)	<0.001*
other subtype 2^d^	57(92)	61(84)	118(88)		15(65)	103(93)	118(88)	
Acinar	30(48)	29(40)	59(43)		7(30)	53(47)	59(44)	
Papillary	8(13)	21(29)	29(21)		5(22)	24(21)	29(21)	
Solid	3(5)	8(11)	11(8)		6(26)	5(4)	11(8)	
Mucinous	1(2)	3(4)	4(3)		2(9)	2(2)	4(3)	
Micropapillary	1(2)	2(3)	3(2)		2(9)	1(1)	3(2)	
Sieve	2(3)	2(3)	4(3)		0(0)	4(3)	4(3)	
Pleural invasion	25(40)	27(37)	52(39)	0.691	9(39)	41(37)	52(39)	0.819

* *P* values were based on comparisons between the two groups

^a^ TNM staging was based on the IASLC 8th TNM Lung Cancer Staging System

^b^ Lepidic predominant includes: adenocarcinoma in situ, minimally invasive adenocarcinoma, and lepidic predominant invasive adenocarcinoma

^c^ Other subtypes1 include: acinar, papillary, micropapillary, and solid predominant adenocarcinoma, as well as variants of invasive adenocarcinoma

^d^ Other subtypes2 include: adenocarcinoma in situ, minimally invasive adenocarcinoma, and lepidic, , acinar, papillary, micropapillary, and sieve predominant adenocarcinoma

*EGFR* epidermal growth factor receptor, *ALK* anaplastic large-cell lymphoma kinase, *EGFR +* EGFR mutation, *EGFR-* EGFR wild type mutation, *ALK +* ALK positive, *ALK-* ALK negative

On a per patient basis, younger patients (49 ± 7 vs 58 ± 7, *p* = 0.002) and those who had never smoked (*p* = 0.018) were more frequently included in the ALK-positive group (n = 13) than in the ALK-negative group (*n* = 54), but no significant association was found with sex (*p* = 0.736), history of smoking (*p* = 1.000) or lymph node metastasis (*p* = 0.110) (Table 2). On a per lesion basis, the present study cohort was divided into an ALK-positive group (*n* = 23) and an ALK-negative group (*n* = 112). ALK-positivity more frequently occurred in tumours with a solid predominant subtype or in mucinous adenocarcinoma (*p* < 0.001). However, no differences were found between the two groups with regard to TNM stage or pleural invasion, as shown in Table 3.

### Interobserver agreement in CT interpretation

The intraclass correlation coefficient for tumour maximum diameter was 0.940 (95% CI: 0.838, 0.978). Regarding other CT features, the concordance between the two observers was good, with the *k* coefficients ranging between 0.640 and 0.950 (Table 4).

**Table 4:** Analysis of inter-reader agreement percent of concordance and kappa of agreement

CT features	N(% of concordance)	Kappa(95%CI)	Kappa interpretation
Shape	125/135	0.846(0.742–0.933)	Almost perfect
Type	131/135	0.950(0.797–0.978)	Almost perfect
Texture	127/135	0.860(0.749–0.948)	Almost perfect
Bubblelike lucency	128/135	0.861(0.754–0.956)	Almost perfect
Margins	121/135	0.759(0.632–0.871)	Almost perfect
Vascular convergence	124/135	0.813(0.698–0.916)	Almost perfect
Air bronchogram	124/135	0.820(0.703–0.921)	Almost perfect
Pleural retraction	133/135	0.969(0.922–1.00)	Almost perfect
Spiculate	130/135	0.923(0.847–0.984)	Almost perfect
Calcifications	128/135	0.640(0.335–0.881)	Substantial
Enhancement degree	89/105	0.634(0.447–0.783)	Substantial
Lymphadenopathy	130/135	0.881(0.766–0.976)	Almost perfect
Cavitation	132/135	0.758(0.393–1.00)	Almost perfect
Heterogeneity	126/135	0.838(0.731–0.924)	Almost perfect
Peripheral fibrosis	127/135	0.881(0.794 ~ 0.956)	Almost perfect
Peripheral emphysema	121/135	0.740(0.605 ~ 0.858)	Substantial
Necrosis	122/135	0.789(0.674–0.893)	Almost perfect

### Correlation of EGFR mutations and ALK rearrangements with CT features

**Fig. 1:**
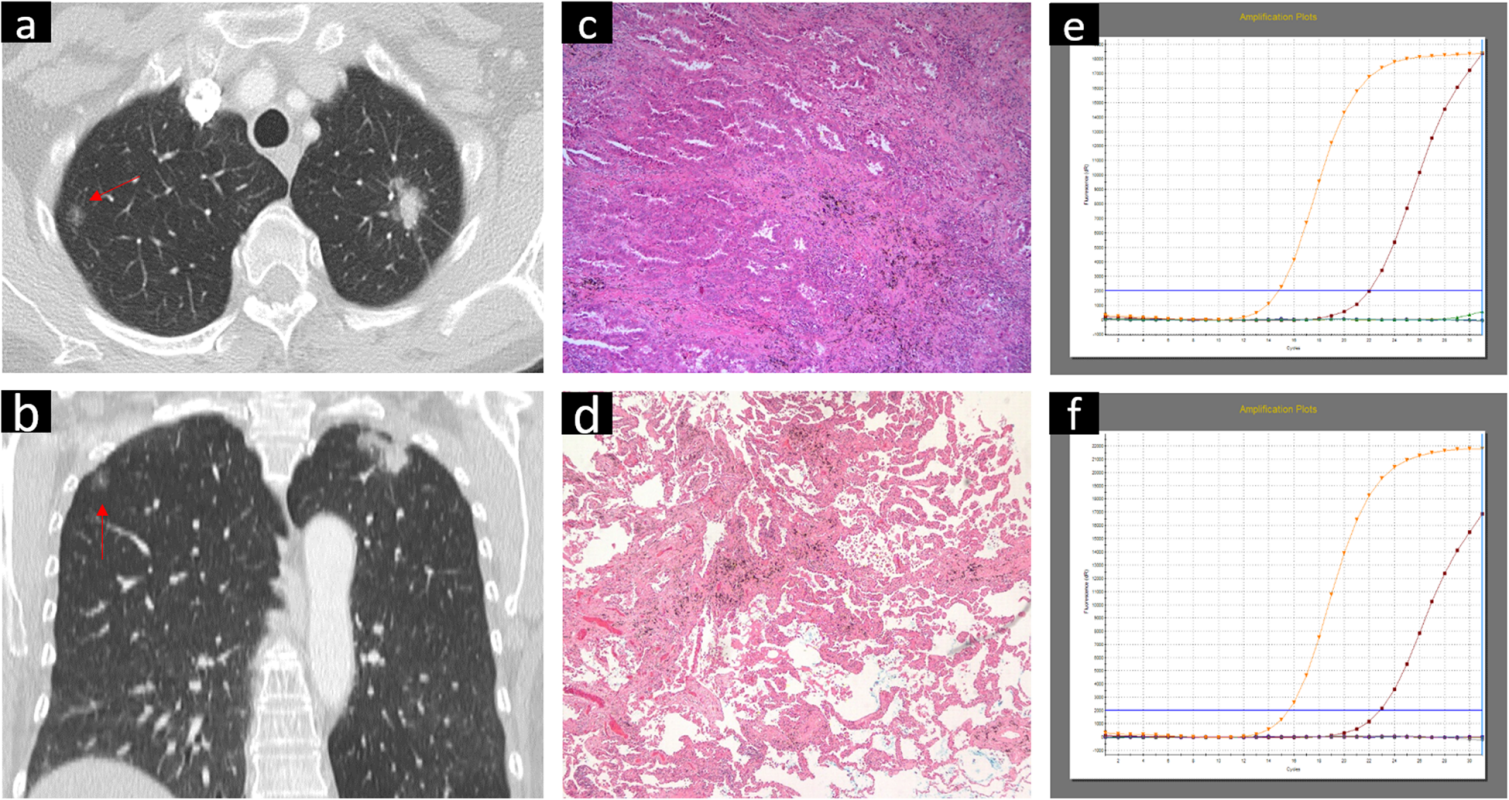
A 62 year-old female with two concurrent primary lung adenocarcinomas, one was located in the apical posterior segment of the left upper lobe (lesion A) (**a**), and the other was located in the right upper lobe (lesion B) (**b**). On CT, lesion A presented as a lobulated, solid nodule with spiculate and pleural traction. Lesion B exhibited light, poorly defined ground glass opacity (GGO). Haematoxylin-eosin (H & E) staining (**c**) showed that lesion A was an invasive lung adenocarcinoma with an acinar predominant subtype, and lesion B was a microinvasive lung adenocarcinoma with a lepidic predominant subtype. The ARMS method (**e**, **f**) revealed a 19_del mutation within exon 19 of the EGFR gene in both tumours

All CT signs were recorded in terms of prelesions: 10.4% (7/67) of the lesions in the same patient were located in the same lung segment, 15% (10/67) were located in the same lung lobe, 59.7% (40/67) were located in the ipsilateral lung, and 23.9% (16/67) were located in the contralateral lung (Fig. 1).

**Fig. 2:**
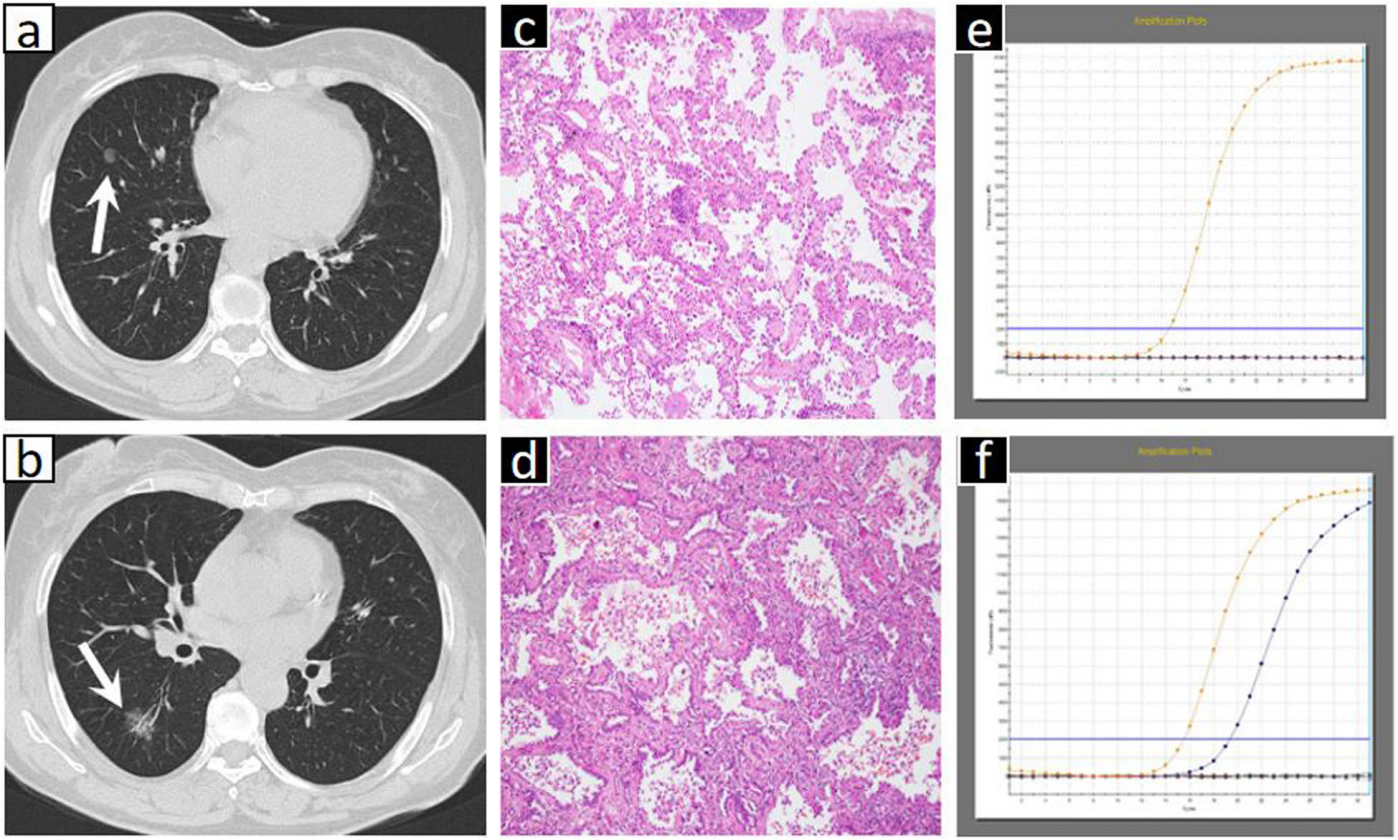
A 63-year-old female with two concurrent primary lung adenocarcinomas, one in the right middle lobe (lesion A) (**a**) and one in the right lower lobe (lesion B) (**b**). By CT, lesion A appeared as a pure ground glass opacity (GGO) nodule, and lesion B exhibited a mixed GGO with a lobulated border. Haematoxylin and eosin (H&E) staining (**c**, **d**) showed different histological adenocarcinoma types, and the ARMS method (**e**, **f**) revealed a 19_del mutation within exon 19 of the EGFR gene in the lower lobe tumour but not in the middle lobe tumour

**Fig. 3:**
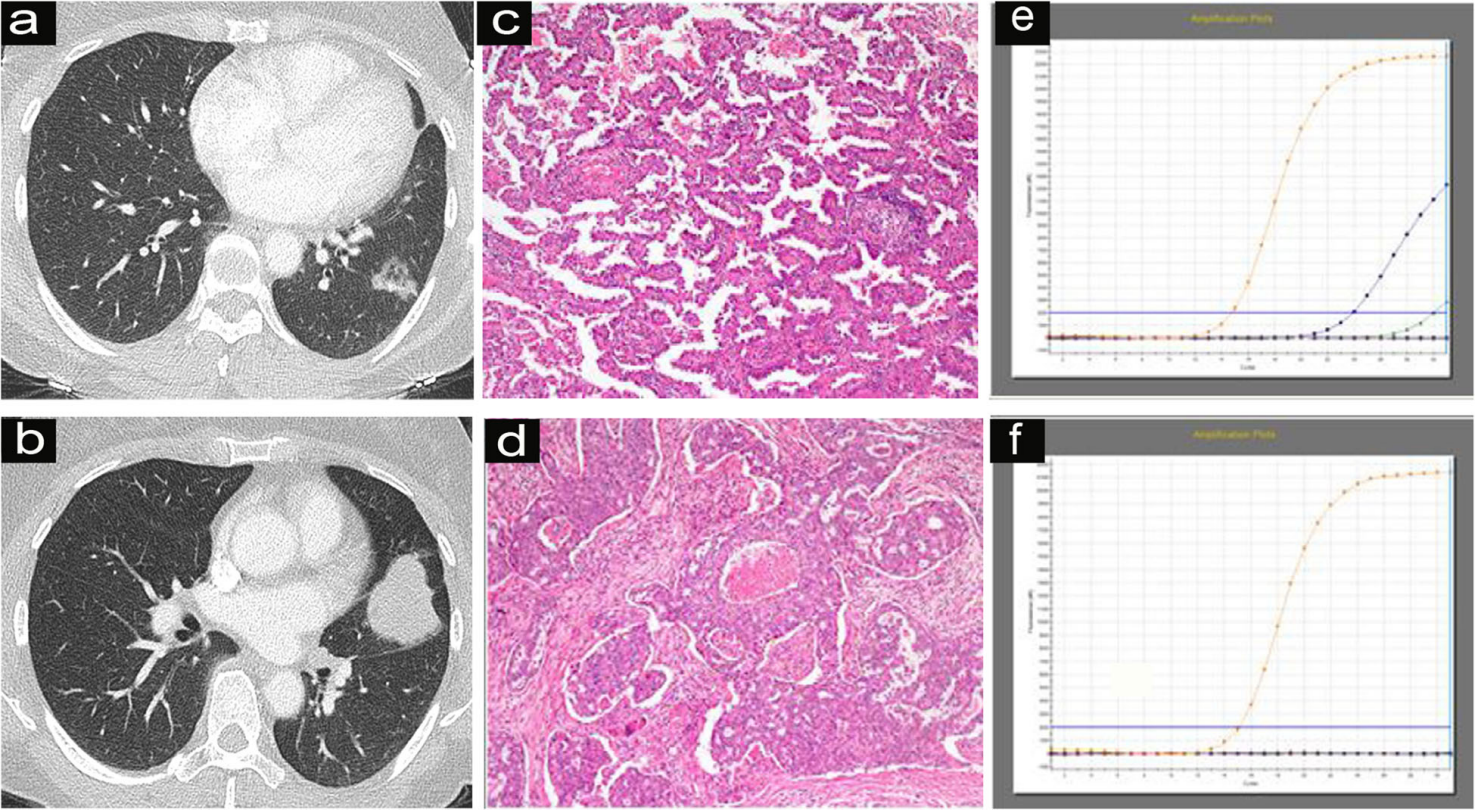
A 56-year-old female with two concurrent primary lung adenocarcinomas, one in the left lower lobe (lesion A) (**a**) and one in the left upper lobe (lesion B) (**b**). Lesion A was a well to moderately differentiated adenocarcinoma and appeared as a mixed GGO nodule on CT. Lesion B was an adenocarcinoma and appeared as a solid mass with a lobulated border on CT. Haematoxylin and eosin (H&E) staining showed (**c**) papillary patterns for T1 but (**d**) solid and cribriform predominant patterns for T2. ARMS-PCR analysis identified an EGFR mutation in (**e**) T1 but not in (**f**) T2. IHC and fluorescence in situ hybridization (FISH) showed positive and negative results, respectively, for ALK in T1 but positive results in T2

Table 5 shows the CT feature comparisons of different EGFR and ALK statuses in patients with MPLAs. The present series revealed that ground-glass opacity (GGO) (*p* = 0.020) (Fig. 1) or mixed GGO (*p*<0.001) (Fig. 2) and air bronchograms (*p* = 0.012) (Fig. 2) were associated with EGFR mutations. No other CT signs were associated with EGFR mutation status (Table 5). Logistics regression analysis showed that GGO (OR = 6.550, *p* = 0.010) was correlated with EGFR mutations, whereas air bronchograms were not (OR = 3.527, *p* = 0.060). In addition, ALK positivity was associated with solid tumours in MPLAs (*p <* 0.004) (Fig. 3). No other CT signs were associated with ALK rearrangement status (Table 5). Logistics regression analysis showed that solid nodules (OR = 6.550, *p* = 0.010) were an independent factor predicting ALK positivity in MPLAs.

**Table 5:** CT features comparison of multiple primary lung adenocarcinomas in different EGFR and ALK status (in pre-lesions)

CT features	EGFR+ (*n* = 62)	EGFR-(*n* = 73)	Total	*P*	ALK+ (*n* = 23)	ALK-(*n* = 112)	Total	*P*
Diameter (cm)^a^	1.7 ± 1.3	1.7 ± 1.6	1.7 ± 1.5	0.835	2.1 ± 2.1	1.6 ± 1.3	1.7 ± 1.5	0.237
Type				0.118				0.305
Central	15(24)	10(14)	25(19)		6(26)	19(17)	25(19)	
Peripheral	47(76)	63(86)	110(81)		17(74)	93(83)	110(81)	
Distribution				0.537				0.007*
left upper lobe	13(21)	12(16)	25(19)		5(22)	20(18)	25(19)	
left lower lobe	12(19)	8(11)	20(15)		1(4)	19(17)	20(15)	
right upper lobe	17(27)	21(29)	38(28)		3(13)	35(31)	38(28)	
right middle lobe	8(13)	12(16)	20(15)		2(9)	18(16)	20(15)	
right lower lobe	12(19)	20(27)	32(23)		12(52)	20(18)	32(23)	
Texture				0.002^*,b^				0.005^*,b^
Solid	35(56)	59(81)	94(70)		22(96)	72(64)	94(70)	
GGO	27(44)	14(19)	41(30)		1(4)	40(36)	41(30)	
pGGO	4(6)	10(14)	14(10)	0.514^c^	0(0)	14(13)	14(10)	0.070^c^
mGGO	23(37)	4(5)	27(20)	<0.001^*,d,e^	1(4)	26(23)	27(20)	0.021*^d,e^
Shape				0.259				0.341
lobulated	20(32)	31(41)	51(38)		7(30)	46(41)	51(38)	
others	42(68)	42(58)	84(62)		16(70)	66(59)	84(62)	
Spiculate	25(40)	28(37)	53(39)	0.816	14(61)	39(35)	53(39)	0.134
Margin definition				1.000				0.453
well-defined	18(29)	21(29)	39(29)		5(22)	34(30)	39(29)	
poor-defined	44(71)	52(71)	96(71)		18(78)	78(70)	96(71)	
Air bronchogram	28(45)	18(25)	46(34)	0.012*	5(22)	41(37)	46(34)	0.171
Heterogeneity	24(39)	35(48)	49(36)	0.358	12(52)	47(42)	49(36)	0.369
Pleural attachment	28(45)	25(34)	53(39)	0.196	8(35)	45(40)	53(39)	0.815
Cavitation	3(5)	2(3)	5(4)	1.000	3(13)	2(2)	5(4)	1.000
Bubble-like lucency	17(27)	15(21)	32(24)	0.349	5(22)	27(24)	32(24)	1.000
Calcifications	5(8)	3(4)	8(6)	0.332	2(9)	6(5)	8(6)	1.000
Necrosis	25(40)	23(32)	48(36)	0.286	6(26)	42(38)	48(36)	0.298
Vascular convergence	15(24)	27(37)	42(31)	0.110	6(26)	36(32)	42(31)	0.298
Peripheral fibrosis	22(35)	21(29)	43(32)	0.404	7(30)	36(32)	43(32)	0.873
Peripheral emphysema	14(23)	20(27)	34(25)	0.521	5(22)	2(2)	34(25)	0.676
Enhancement degree				0.807				0.559
mild	34(69)	41(73)	75(71)		16(70)	59(70)	75(71)	
moderate	12(24)	13(23)	25(24)		6(26)	19(23)	25(24)	
marked	3(7)	2(4)	5(5)		1(4)	4(7)	5(5)	

* *P* values<0.05

^a^ The maximum diameter of the lesion (in centimeters) evaluated on the multiplanar reconstructions (MPRs) with a soft tissue window;

^b^ Comparison between solid and GGO

^c^ Comparison between solid and pGGO

^d^ Comparison between solid and mGGO

^e^ Comparison between pGGO and mGGO

*EGFR+* epidermal growth factor receptor mutation, *EGFR-* EGFR wild-type group, *GGO* ground-glass opacity, *pGGO* pure ground-glass opacity, *mGGO* mix ground-glass opacity

### ROC curve analysis

**Fig. 4:**
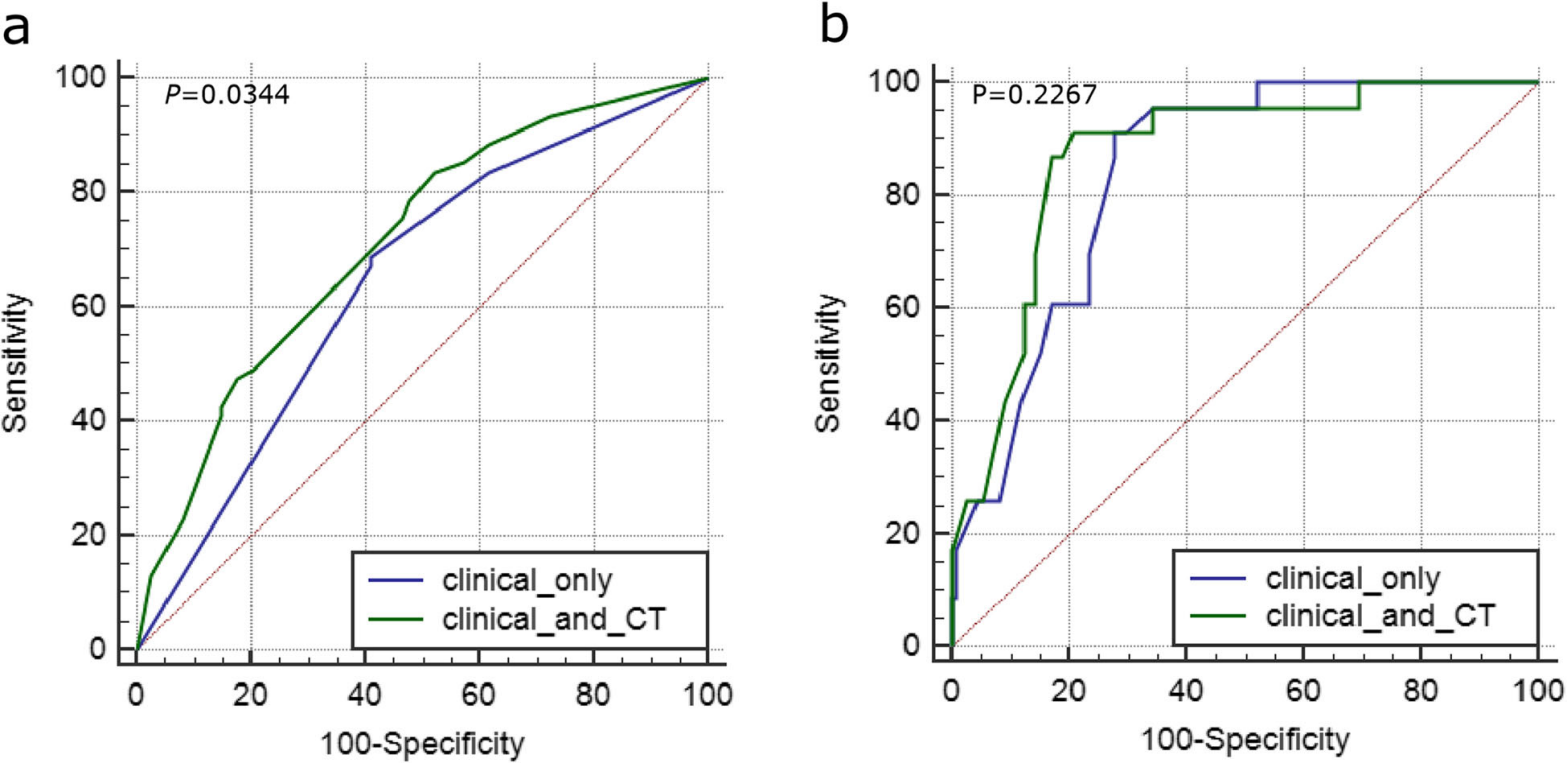
Receiver operating curve (ROC) curve for EGFR mutation (**a**) or ALK positivity (**b**) prediction in MPLAs using clinical features alone or combined with CT signs

For prediction of EGFR mutations, receiver operating characteristic curve (ROC) analysis yielded area under the curve (AUC) values of 0.647 and 0.712 for clinical-only or combined CT features, respectively, and a significant difference was found between them (*p* = 0.344) (Fig. 4a). For prediction of ALK positivity, the ROC curve yielded AUC values of 0.767 and 0.804 for clinical-only or combined CT features, respectively, but no significant difference was found between them (*p* = 0.2267) (Fig. 4b).

## Discussion

In this study, we found that the incidence of MPLAs in our hospital from January 2014 to February 2019 was 5.6%, which is consistent with previous reports (0.2 to 8%) [3–5]. The average age of patients with MPLAs in our study was 58 ± 7 years old, and the proportion of women was significantly higher than that of men (2:1), similar to a previous study [8]. In terms of patients, the effective EGFR mutation rate in MPLA patients was 64.2%, which was higher than that in single lung adenocarcinoma patients (27–56%) [26–28]. This result may be because EGFR mutations in an MPLA patient were defined as at least one lesion harbouring an EGFR mutation, which would overestimate the EGFR mutation rate. When based on lesions, the EGFR mutation rate was 45.9%, which was consistent with the results of previous studies [24–26].

When based on patients, EGFR mutations in the MPLA group were more common in women without a smoking history than in the wild-type EGFR group, similar to the clinical characteristics of patients with EGFR mutations in single primary lung adenocarcinoma (SPLA) [26–28]. When based on lesions, no significant difference in the TNM stage of tumours was found between the EGFR mutation group and the wild-type EGFR group, in contrast with the results of Tu et al. [27]; they found that the EGFR mutation rate in early NSCLC patients was significantly higher than that in advanced NSCLC patients. This difference may be because most of the patients in our study were stage III-IV (68.9%), while in the Tu et al. series, stage III-IV lung adenocarcinoma only accounted for 22.6% of the patients. On the other hand, we found that EGFR mutation was less associated with lymph node metastasis, supporting a lower stage in EGFR mutation cases. Previous studies reported that the mutation rate of EGFR in lepidic predominant lung adenocarcinoma was higher than that in adenocarcinoma of other subtypes [16, 26, 29]. In this study, although the EGFR mutation rate in adenocarcinoma with the lepidic predominant subtype (24.2%) was higher than that in other subtypes (13.7%), there was no significant difference between them, likely because the incidence of the lepidic predominant subtype was low (18.5%).

In the present study, most lesions were located in the ipsilateral lung (59.7%) and in the same lung lobe and the same lung segment, followed by the contralateral lung (23.8%). However, Arai et al. [30] demonstrated that 50% of double primary cancers were located in the contralateral lung, while intrapulmonary metastatic lesions mainly occurred in the same lobes (84.2%). The studies may differ because all the tumours included in our cases were surgically removed. In terms of tolerance, patients exhibited better surgery tolerance when the lesions were located in the ipsilateral lung. We excluded some lesions in the contralateral lung that had not been operated on. Moreover, in the present study, some patients with inoperable lesions are still being followed up.

Previous studies [14, 17, 18, 26, 31] have analysed the potential relationship between the imaging signs of SPLA and EGFR mutation. Among CT features, most of the studies concentrated on the correlation between GGO and EGFR mutation. The present study found that EGFR mutations more frequently showed GGO in CT, consistent with the results of most previous studies in SPLA [14, 15, 26]. This finding may be due to the inverse relationship between EGFR replication and the percentage of GGO on CT [32, 33]. In addition, in our study, mixed GGO (mGGO) lesions were more susceptible to EGFR mutations than pure GGO (pGGO) lesions. A possible explanation for the above phenomenon may be that EGFR mutations can promote the conversion of pure GGO to mixed GGO [34]. In addition, this study revealed that EGFR mutations in MPLAs are more common in air bronchograms on CT, which is consistent with previous studies in SPLA [14, 19]. The air bronchograms showed that certain tumours had not yet invaded the bronchus, suggesting a weak aggressiveness of tumours with EGFR mutation. However, no correlation was found between other CT features and EGFR mutations in MPLA lesions, such as tumour size [16], lobulation [14], spicules [15], and pleural attachment [19], as detected in SPLA. These differences occurred not only in our series and previous studies but also differ between previous studies. Possible explanations for the above differences are the different study designs and the demographic features. ROC curve analysis for predicting EGFR mutation showed that the use of clinical combined CT features was significantly superior to use of clinical variables only. Therefore, we may reasonably consider that MPLA tumours with EGFR mutations have imaging patterns similar to those of single lung adenocarcinomas, which emphasizes the need to apply CT features to predict EGFR mutation in MPLA lesions that cannot be biopsied.

ALK positivity has been identified in 0.4 to 13.5% of unselected NSCLC patients [35, 36]. In this study, patients with MPLAs had a slightly higher ALK-positive rate (17.0%), which may be due to ALK positivity being more frequently found in advanced lung adenocarcinoma [37]. Most of the MPLA patients in our study had high TNM stages (III-IV, 86.6%), indicating that ALK-positive status was related to advanced tumour stage. Previous studies have reported that ALK-positive patients tend to be younger and are more often never-smokers than patients with non-ALK rearrangement [38]; this was also confirmed in our study. A recent study by Li et al. [39], conducted with a relatively large sample, demonstrated that ALK positivity was more common in the solid predominant subtype of adenocarcinoma. Akihiko et al. [40] reported that NSCLC with ALK positivity commonly exhibits solid or acinar growth patterns, sieving structures, mucous cells (sign or ring cells) and abundant extracellular mucus. Similarly, our study found that the rate of ALK positivity is higher in the solid predominant subtype or in mucinous adenocarcinoma.

Regarding CT imaging, previous studies [19, 20, 31, 41] have demonstrated that a larger volume, solid mass, extensive lymph node metastasis, pleural invasion, pleural effusion and distant metastasis are associated with ALK positivity, suggesting a highly aggressive feature. In our series, ALK-positive MPLAs were independently associated with the manifestation of solid nodules or masses without a GGO appearance, in line with the above report [20, 31]. This can be explained by the pathological results in the present study showing that ALK positivity was related to the solid predominant subtype or mucinous adenocarcinoma, because adenocarcinoma with the solid predominant subtype or mucinous adenocarcinoma hardly presented with GGO on CT. Additionally, Aritoshi et al. [42] found that the prognosis was better for double primary lung cancer patients with two GGO nodules than for those with two solid nodules. These findings indicate that MPLA patients who are ALK positive may have a poor prognosis. However, no correlation was found between other CT features and ALK positivity, which may be due to the small sample size in the present study. For prediction of ALK positivity, the ROC curve yielded AUC values of 0.767 and 0.804 for clinical-only or combined CT features, respectively, but no significant difference was found between them. This result may be because few CT features are related to ALK positivity in the present series, which provides a limited diagnostic value for ALK positivity. However, we believe that more promising results will be found in future in studies with a larger sample.

Our study has limitations. 1) The study was performed at a single centre with a small series of patients, especially in terms of the number of ALK-positive cases (13 patients; 23 lesions); Thus, multicentre studies with a large sample size are needed to verify the conclusions of this study and the subgroup analysis. 2) CT features can not only be used to predict the gene status of MPLAs but also to follow up patients with MPLAs, because these patients will develop more lesions that we cannot be biopsied. However, our cases were collected from 2014 to 2019; therefore, the follow-up time was insufficient. Hence, the follow-up chest CT results were not analysed here. Future long-term studies were required to explore the outcome of these patients whether they behaved like true synchronous T1/T2 or closer to T1NxM1a. 3) Third, the present study analysed only adenocarcinoma and did not include other histologic subtypes. However, this is understandable, as the majority of EGFR mutations and ALK-positivity cases were found in adenocarcinomas, with an extremely low mutation rate in squamous cell carcinoma (< 5%) [43]. 4) To ensure the reliability of the obtained pathological samples, all cases included in this study were surgically resected. In addition, we excluded some lesions that were only confirmed by biopsy but were not surgically resected, which inevitably affected the comprehensiveness of the study results. 5) The relationship between CT findings and different EGFR mutation types (exon 18,19,20,21) was not discussed in our study due to the small sample, and Lee et al. [16] demonstrated that the GGO volume percentage was significantly higher in tumours with exon 21 mutations than in tumours with other EGFR mutations. However, our results provide directions for further research.

## Conclusion

In conclusion, among patients with MPLAs, nonsmoking women with less lymph node metastasis and patients who present with GGO and air bronchograms on CT are more susceptible to EGFR mutations. In nonsmoking patients, young patients with solid lesions on CT were recommended for the ALK status test. Therefore, our study can reasonably conclude that MPLA tumours with EGFR mutations and ALK positivity have imaging patterns similar to single lung adenocarcinomas. These results can guide clinical treatment of different nodules in patients with MPLAs and aid in development of the best treatment strategy for those patients. However, because this is a small sample, retrospective study, confirmation of these conclusions in larger prospective studies is needed.

## Data Availability

The datasets used and/or analysed during the current study are available from the corresponding author on reasonable request.

## Abbreviations

MPLCs: Multiple primary lung cancers
MPLAs: Multiple primary lung adenocarcinomas
SPLA: Single primary lung adenocarcinoma
CT: Computed tomography
EGFR: Epidermal growth factor receptor
ALK: Anaplastic lymphoma kinase
NSCLC: Non-small cell lung carcinoma
TKIs: Tyrosine kinase inhibitors
GGO: Ground-glass opacity
pGGO: Pure GGO
mGGO: Mixed GGO
PFS: Progression-free survival
TNM: Tumour node metastasis
CHA: Comprehensive Histologic Assessment
IASLC: International Association for the Study of Lung Cancer
MDT: Multidisciplinary team
MDT: Multidisciplinary teams
ROC: Receiver operating characteristic
AUC: Area under the curve
ARMS: Amplified drug resistance mutation system
IHC: Immunohistochemistry
FFPE: Formalin-fixed paraffin-embedded
FISH: Fluorescence in situ hybridization
